# Gluten Contamination in Naturally or Labeled Gluten-Free Products Marketed in Italy

**DOI:** 10.3390/nu9020115

**Published:** 2017-02-07

**Authors:** Anil K. Verma, Simona Gatti, Tiziana Galeazzi, Chiara Monachesi, Lucia Padella, Giada Del Baldo, Roberta Annibali, Elena Lionetti, Carlo Catassi

**Affiliations:** 1 Celiac Disease Research Laboratory, Department of Pediatrics, Università Politecnica delle Marche, 60123 Ancona, Italy; simona.gatti@hotmail.it (S.G.); t.galeazzi@univpm.it (T.G.); chiara.monachesi28@gmail.com (C.M.); luciapadella@libero.it (L.P.); mariaelenalionetti@gmail.com (E.L.); c.catassi@univpm.it (C.C.); 2 Department of Pediatrics, Università Politecnica delle Marche, 60123 Ancona, Italy; giadadelbaldo@gmail.com (G.D.B.); robertannibali@hotmail.it (R.A.)

**Keywords:** celiac disease, gluten-free products, naturally gluten-free, R5 ELISA, oats, buckwheat, lentils

## Abstract

Background: A strict and lifelong gluten-free diet is the only treatment of celiac disease. Gluten contamination has been frequently reported in nominally gluten-free products. The aim of this study was to test the level of gluten contamination in gluten-free products currently available in the Italian market. Method: A total of 200 commercially available gluten-free products (including both naturally and certified gluten-free products) were randomly collected from different Italian supermarkets. The gluten content was determined by the R5 ELISA Kit approved by EU regulations. Results: Gluten level was lower than 10 part per million (ppm) in 173 products (86.5%), between 10 and 20 ppm in 9 (4.5%), and higher than 20 ppm in 18 (9%), respectively. In contaminated foodstuff (gluten > 20 ppm) the amount of gluten was almost exclusively in the range of a very low gluten content. Contaminated products most commonly belonged to oats-, buckwheat-, and lentils-based items. Certified and higher cost gluten-free products were less commonly contaminated by gluten. Conclusion: Gluten contamination in either naturally or labeled gluten-free products marketed in Italy is nowadays uncommon and usually mild on a quantitative basis. A program of systematic sampling of gluten-free food is needed to promptly disclose at-risk products.

## 1. Introduction

Celiac disease (CD) is an autoimmune condition characterized by permanent intolerance to dietary gluten, a protein complex found in wheat, rye and barley, occurring in genetically predisposed individuals [1]. The hallmarks of active CD are the presence of serum autoantibodies (e.g., IgA antitransglutaminase and antiendomysial antibodies) and a small intestinal enteropathy characterized, in typical cases, by villous atrophy, crypt hypertrophy and increased number of intraepithelial lymphocytes (IELs). Treatment of CD is based on the lifelong exclusion of gluten-containing food from the diet. The gluten-free diet (GFD) determines the gradual disappearance of symptoms and serum autoantibodies, and the normalization of the intestinal histological architecture [2].

Unfortunately, CD patients are highly sensitive to the toxic effect of gluten. It has been shown that the protracted ingestion of traces of gluten (10–50 mg on a daily basis) may damage the integrity of the small intestinal mucosa, an increased number of IELs being the first marker of mucosal deterioration [3]. By combining these toxicity data with the observed food intake, it has been calculated that gluten-free products with less than 20 mg/kg (or parts per millio*n* = ppm) of gluten contamination are safe over a wide range of daily consumption [4]. The 20 ppm threshold for gluten-free food has been endorsed by the Codex Alimentarius [5] and other agencies, e.g., the US Food and Drug Administration (FDA) and the European Food Safety Authority (EFSA) [6]. 

Despite the availability of a wide range of natural (by origin) and industrially-prepared gluten-free food, complete avoidance of gluten from the diet is difficult to maintain. Gluten is indeed a “pervasive” nutrient that may contaminate otherwise gluten-free items along the production chain, from the field to the milling, stockage and manufacture steps [7]. Furthermore wheat flour or purified gluten are largely added by the food industry to naturally gluten-free food, due to its technological properties, particularly the high visco-elasticity. Protracted intake of items contaminated with gluten traces may cause persistent intestinal damage and symptoms in treated CD patients [8]. 

The scarcity of published data on the possible gluten contamination of nominally gluten-free products is a matter of concern. This is the reason why we decided to undertake the current study, by measuring gluten in a large sample of gluten-free products that are currently on the market in Italy, using the only method (R5 ELISA) approved by the EU regulation. We present here the final results of these analyses on 200 commercially available gluten-free products.

## 2. Materials and Methods

### 2.1. Collection of Food Products

A sampling plan was developed to analyze gluten-free products, including substitutes of wheat-based food, and other starch-rich food, e.g., legumes, that are extensively used in day-to-day meal preparation by individuals following a gluten-free diet. Selected products included different brands of (a) gluten-free flour, pasta, snacks, cookies, muesli, breakfast cereals, bread, and pizza; (b) rice, oats, buckwheat, quinoa, amaranth, mixed cereals, lentils, and chickpeas. Between April and October 2016, a total of 200 commercially available food products of common use were purchased in randomly chosen supermarkets in Ancona, Italy. 

**Figure 1:**
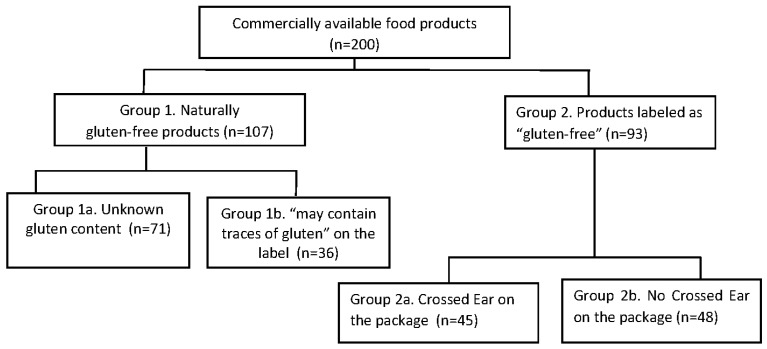
Types of food products analyzed in this study.

Food products were carefully identified and categorized into two broad categories, i.e., naturally (by origin) gluten-free products (Group 1) and labeled “gluten-free” products (Group 2). Group 1 was further divided into 1a, reporting no information of gluten content (defined herein as **“**products with unknown gluten content”)**,** and 1b, reporting “may contain traces of gluten” on the label. Group 2, i.e., certified gluten-free products, were categorized as 2a, including products fulfilling the EU regulation for gluten-free products (UE 828/2014) plus the quality certification released by the Italian Celiac Society (identified by the “Crossed Ear” symbol on the package) or 2b, including gluten-free products fulfilling the EU regulation for gluten-free products only (Figure 1). 

### 2.2. Determination of Gluten Content

All food products were subjected to gluten content determination by the Ridascreen Gliadin sandwich R5 enzyme-linked immunosorbent assay R-7001 (R-Biopharm, Darmstadt, Germany) at the Celiac Disease Research Laboratory of the Department of Pediatrics, Università Politecnica delle Marche, Ancona, Italy. During each run of ELISA, manufacturer’s guidelines were strictly followed. Briefly, the steps of the ELISA procedure were as follows. 

#### 2.2.1. Extraction and Preparation of Samples

All samples were given a unique laboratory code and their details (including brand, cost, ingredient, food type, etc.) were recorded on an Excel sheet. Five grams of each sample were homogenized and crushed in a laboratory blender (solid food products). Each time after the crushing of a particular sample, parts of the blender were removed and washed with alkaline-enzyme detergent and rinse with 70% ethanol and dried before processing of another sample. Homogenized samples were stored in sterile tubes. Ridascreen R-7006 cocktail solution, containing detergents and reducing agent, was used for the extraction of samples. One-quarter gram of processed solid samples and 0.25 mL of liquid samples were measured in separate pre-labeled falcon tubes. In tannin and polyphenol containing products additionally 0.25 g of skimmed milk powder was added. After this preparation, 2.5 mL of cocktail solution was added in each tube under a chemical hood and tubes were vortexed and kept in water bath at 50 °C for 40 min. After the incubation, tubes were allowed to maintain room temperature and 7.5 mL of freshly prepared 80% ethanol was mixed in each tube and kept on a shaker for 1 h. Samples were then transferred into 1.5 mL of Eppendorf tubes and centrifuged at least 2500 g for 10 min, supernatant was separated and collected into another 1.5 mL Eppendorf tube and stored at room temperature. To avoid any possible cross contamination, samples were crushed in different rooms and at different time intervals. 

#### 2.2.2. Gluten Quantification

Extracted food samples were diluted at 1:12.5 in provided sample dilution, standard and samples were added in duplicate into pre-defined ELISA wells and enzyme conjugate was added to each well followed by wash of ELISA plate by washing buffer and kept for incubation for 30 min at room temperature. Substrate and chromogen were added and the reaction was stopped by provided stop solution and reading was obtained at the absorbance of 450 nm. Samples that showed an absorption value above the highest standard value were further diluted to get the absorption value within the range. The lower limit of the quantification was 2.5 ppm (mg/kg) of gliadin, corresponding to 5 ppm (mg/kg) of gluten. Results were calculated by the suggested method and then entered in the Excel sheet. 

Food products containing gluten level lower than 20 ppm were considered as gluten-free while products with gluten level between 20 and 100 ppm were classified as products with low gluten contamination and products with more than 100 ppm of gluten were considered significantly contaminated. All products with a gluten level higher than 20 ppm were re-extracted and analyzed second time.

### 2.3. Quality Control

Each time absorption value of ELISA standards was assured with the quality assurance certificate provided with the ELISA kit. The result of each run was discussed with research group members and random results were sent to the principal company for expert comments and suggestion. At different time intervals, all the group members gathered and discussed the procedure and further action.

### 2.4. Cost Analysis: Correlation between the Cost of the Product and Gluten Contamination

If at least 5 products with similar ingredients from different brands were available, the mean price was calculated. Then, for each product the price index (PI) was calculated as the product price divided by the mean price of the category. The PI was then categorized in 3 groups (price categories): PI < 0.75 (products with a low price), PI: 0.75–1.25 (products with an average price), PI > 1.25 (products with a high price).

### 2.5. Statistics

Data are presented as proportions, means and S.D., medians and range, as appropriate. The Kruskal-Wallis one-way analysis of variance was used to determine if there was statistically significant difference of gluten contamination between the four groups of products (1a, 1b, 2a, and 2b), and, if significant, post hoc test was used for multiple comparisons. Comparison between proportions of contaminated (>20 ppm) and not contaminated (<20 ppm) samples within each group was calculated by the Fisher’s test. Spearman’s test was used to correlate quantitative variables (prices and gluten levels). Results were found significant when *p* < 0.05. The statistical analysis was performed using the Software Program Stata System (SPSS) v17.0 (Chicago, IL, USA).

## 3. Results

### Detection of Gluten Contamination 

Overall, 200 food products were analyzed: 107 in Group 1 (group 1a, *n* = 71; group 1b, *n* = 36) and 93 in Group B (group 2a, *n* = 45; group 2b, *n* = 48). Overall 173 (86.5%) products were detected with gluten level lower than 10 ppm, nine (4.5%) products contained between 10 and 20 ppm of gluten, and 18 (9%) products were detected with gluten level above the maximum tolerable of 20 ppm (15 products containing less and three products more than 100 ppm of gluten) (Table 1). 

**Figure 2:**
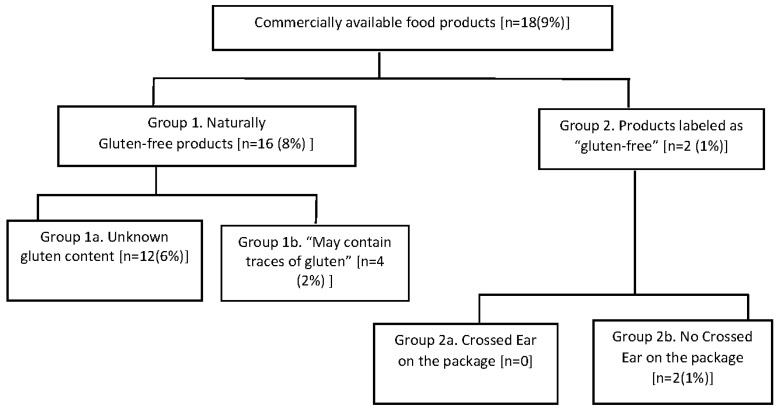
Number of food products containing gluten >20 ppm in different groups.

**Figure 3:**
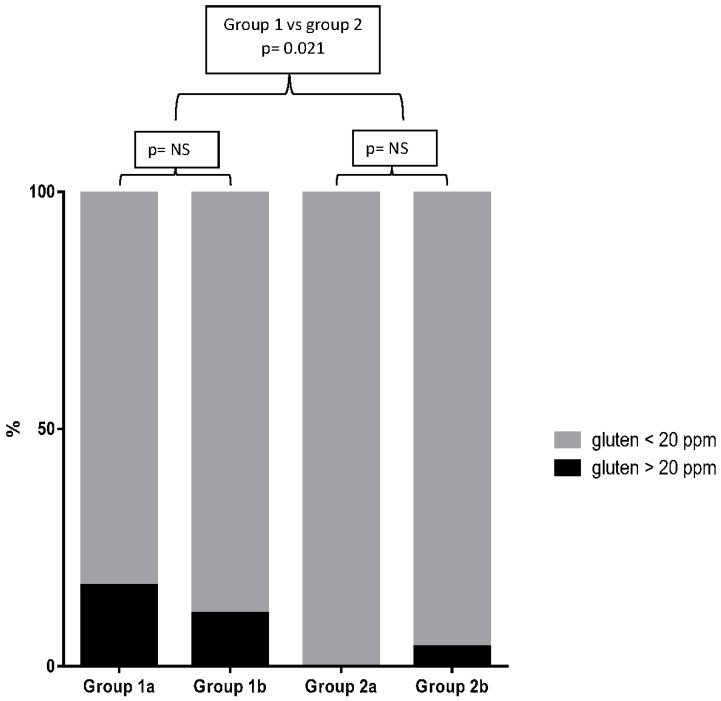
Percentage of contaminated products in each food group.

The proportion of contaminated products (gluten > 20 ppm) according to the staple ingredient and to the food category is reported in Table 2 and Table 3, respectively. In products belonging to group 1, 16 items (8%) were contaminated with more than 20 ppm of gluten, 12 (6%) from sub-group 1a (gluten content unknown) and four (2%) products from sub-group 1b (“may contain gluten”) products. In group 2 (products labeled as gluten free), only two (1%) products were found to have gluten level higher than 20 ppm. These products belonged to subgroup 2b, whereas no “Crossed-Ear” product was found to contain gluten at 20 or more ppm (Figure 2). Overall, we found a significant different proportion of contamination between the four groups of products (Kruskal-Wallis, *p* < 0.01). By multiple comparison, a significant higher proportion of contaminated products was found in group 1a as compared to group 2a (16% vs. 0, *p* < 0.01) (Table 2). No significant difference was found in the proportion of contaminated products between groups 1a and 1b and between groups 2a and 2b, respectively (Table 2, Figure 3). By comparing the staple ingredients, we found a significant higher proportion of contaminated products in oats, buckwheat and lentils as compared to chickpeas, corn, mixed seeds, quinoa, and chocolate. By comparing the food categories, the lunch/dinner products were significantly more contaminated as compared to snacks. 

**Figure 4:**
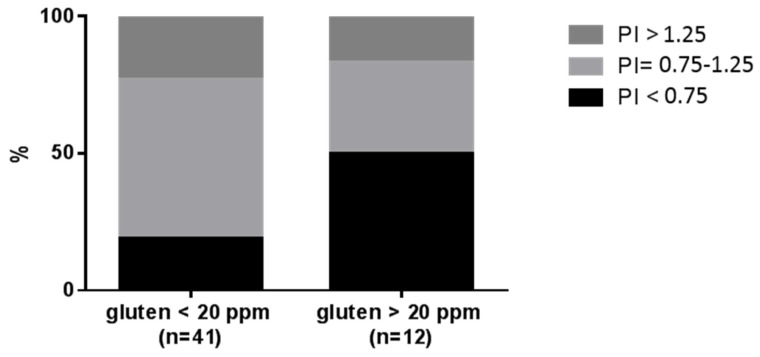
Distribution of price indexes (PIs) according to the level of gluten contamination.

Overall 53 products belonging to six different food categories (lentils, chickpeas, beans, oats, buckwheat and quinoa) were considered for the cost analysis. The PI was not significantly correlated to the content of gluten (*r* = −0.009; *p* = 0.51). However, a significantly different distribution of price categories was found according to the level of gluten contamination. As shown in Figure 4, a higher proportion of low price foods were found in products with levels of gluten > 20 ppm (*p* < 0.01).

## 4. Discussion

Our large survey shows that gluten contamination is low in gluten-free food marketed in Italy, both in terms of the percentage of contaminated products (9%), and the amount of gluten in contaminated products (almost exclusively in the range of the low gluten content 20–100 ppm). However, naturally (by origin) gluten-free products are at significantly higher risk of contamination as compared to products certified as gluten-free; indeed, we found that 16% of naturally gluten-free products with unknown gluten content are contaminated with respect to none of the certified gluten-free products with the crossed-ear symbol. Of note, among the certified gluten-free products without crossed-ear symbol we found that two out of 48 (4%) were contaminated with respect to none of the products with crossed-ear symbol; although this difference is not significant, it may suggest that the more stringent controls performed on “Crossed Ear” gluten-free products guarantee less risk of gluten contamination. 

Our findings are more encouraging than previous studies in some American countries, e.g., contamination was found in 20.5% of gluten-free products marketed in the USA [7] and 21.5% in Brazil [9] respectively, and are in line with previous data from Canada [10] and Europe [4]. Compared to the past, the picture has clearly improved, most likely due to the worldwide implementation of the 20 ppm maximum threshold of gluten contamination established by the Codex Alimentarius in the year 2008 [5]. Based on these data and the dietary habits of the Italian population, the safety threshold of 10–50 mg of daily gluten would hardly be exceeded even by CD patients consuming very large quantities of gluten-free items (provided that no other contaminated food is eaten at the same time).

Quantification of gluten in food is difficult, for several reasons. Firstly gluten is not a single protein but a mix of different protein components (microheterogeneity) generally classified as gliadins, glutenins, globulins and albumins [11]. Measuring all these different fractions is clearly unpractical. Gliadins are the major component on a quantitative basis, and it is generally assumed that the ratio between gliadins (the fraction that is measured with the R5 test) and overall gluten is 1:2 (50%) [12,13]. Other analytical problems include the difficulty in (a) specifically quantifying all the different celiac-toxic peptides contained in gluten; (b) extracting gluten from the different food matrixes; and (c) measuring hydrolysed gluten peptides (e.g., in fermented food such as beer). Several tools have been developed for gluten quantification in food, such as the R5, the G12 and the α-20 antibody-based ELISA kits [14,15]. In the present study we used the R5 method, an ELISA test based on specially designed monoclonal antibodies raised against a pentapeptide from rye. It detects prolamins of wheat (gliadins), rye (secalins) and barley (hordeins), i.e., all the cereals that are toxic for celiac patients, in both raw (flours) and processed food products [16]. It is the only method certified by the Association of Officials Analytical Chemists (AOAC), and is considered as the method of choice for gluten detection in food, according to the Codex Alimentarius Commission and other International Agencies [5,6]. Most recent studies on gluten contamination have been performed using the same R5 test, a finding that allows comparisons among the results of different surveys [4,7,9,10,17].

As reported in previous studies [4,7,9,10,17], we found that products at significant higher risk of contamination of gluten are oats (four out of five examined items), buckwheat (5/12) and lentils-based (4/17) products. Several studies have shown that medium-high amounts of gluten-uncontaminated oats can be safely ingested by patients with CD [18,19]. Official recommendations acknowledge the safety of products containing purified oats, and several national associations for CD allow inclusion of oats in the diet of people with CD [19]. Unfortunately, the commercial oat supply is often contaminated with wheat. In Canada 88% of 133 oat samples were contaminated above 20 ppm [10]. There are possibilities for cross-contamination in the field, in the transport of the grain, in the storage of the grain, and in the milling and packaging facilities [7,10]. This is a deplorable situation since oats is rich in soluble dietary fiber, vitamins and minerals, and may unquestionably improve the nutritional value and increase the palatability of the GFD, while expanding food choices and ultimately improving the life quality of people with CD. Buckwheat is a gluten-free pseudocereal that belongs to the Polygonaceae family. Buckwheat grain is a highly nutritional food component that has been shown to provide a wide range of beneficial effects. Health benefits attributed to Buckwheat include plasma cholesterol level reduction, neuroprotection, anticancer, anti-inflammatory, antidiabetic effects, and improvement of hypertension. In addition, buckwheat has been reported to possess prebiotic and antioxidant activities [20]. The possible gluten contamination of buckwheat has been correlated with the high content of fiber [21]. The frequent gluten contamination of lentils was somewhat unexpected, since this food is a legume and not a cereal, and its production chain is far different from wheat. Lentils are an edible pulse that is part of the human diet since the Neolitic age, being one of the first crops domesticated in the Near East. Lentils are a rich source of numerous nutrients, including protein, starch, folate, thiamin, pantothenic acid, vitamin B6, phosphorus, iron and zinc [22]. The origin of gluten contamination of lentils remains unclear. Many patients or caregivers check lentils seed by seed, and have reported that rare wheat seeds can be found mixed with lentils, most likely due to contamination occurring in the field. The practice of inspecting and washing lentils before cooking should be recommended when the package does not report any gluten-free labeling. 

It is important to underscore that oats, buckwheat and lentils are nutritious dietary components that may increase the variety of carbohydrate- and fiber-rich food in the gluten-free diet. For this reason, we hope that the food industry will pay more attention in ensuring and certifying a gluten-free food chain for these important ingredients. 

Finally, in the present study we also aimed to evaluate if the gluten contamination is, to some extent, related to the cost of the product. It is worth noting that we found that a higher proportion of low price foods were contaminated with respect to higher price foods (*p* < 0.01), suggesting that the lower the price the lower the quality of control on the gluten contamination.

Despite the GFD, many treated CD patients frequently show incomplete resolution of the histological intestinal damage at the follow-up intestinal biopsy, suggesting ongoing gluten ingestion [8]. Since our data and other surveys [4] found that gluten contamination of wheat substitutes does not represent a big issue in recent years, this persistent enteropathy is probably related to different sources of contamination, such as voluntary dietary transgressions, particularly in adolescents, or contaminated meals consumed outside home. Consumption of food prepared away from home plays an increasingly large role in the diet. In the US in 1970, 25.9 percent of all food spending was on food away from home; by 2012, that share rose to its highest level of 43.1 percent (data of the US Department of Agriculture, 2016; www.ers.usda.gov). In restaurants, pizzerias and cafeterias the chance of getting gluten-contamined GF food is higher than home, due to inadequate personnel training, careless use of tools/workbench and so forth. An active policy of training and education on the requirements for the GFD should be addressed to employees at food services.

## 5. Conclusions

Gluten contamination in either naturally or commercial gluten-free products marketed in Italy is nowadays uncommon and usually mild on a quantitative basis. Crossed Ear and higher cost gluten-free products are in general safer than other products. Caution is however needed to interpret these findings, due to the intrinsic limitations of the analytical method for determining gluten traces in food matrixes. A program of systematic sampling of gluten-free food is needed to promptly disclose at-risk products, to ensure the safety of available products and ultimately improve the long-term wellbeing of individuals affected with CD or other gluten-related disorders. 

## Figures and Tables

**Table 1:** Level of gluten contamination in the 200 examined products.

Gluten Content (ppm)	Number of Products	Median (Range) (ppm)	Mean ± SD (ppm)
<10	173	<5 (<0.5–9.3)	n.a.
10–20	9	13.9 (10.4–17.1)	14.1 ± 2.2
>20	18	31.7 (20.4–126.2)	49.2 ± 35.9

n.a. = not applicable (due to the (<5) values).

**Table 2:** Proportion of items containing >20 ppm of gluten by staple ingredient (contaminated/tested products).

Item	Overall *	Group 1		Group 2		*p*	*p*
		Naturally Gluten Free Products		Products Labeled as “Gluten Free”			
		Group 1a	Group 1b	Group 2a	Group 2b	Kruskal-Wallis	Multiple Comparisons
Amaranth	0/2	0/1	0/1	-	-	1.000	
Buckwheat	5/12	3/5	1/3	0/1	1/3	0.695	
Chickpeas	0/6	0/4	0/2	-	-	1.000	
Chocolate	0/9	0/1	0/3	-	0/5	1.000	
Coconut	1/3	1/2	-	-	0/1	0.480	
Corn	0/40	0/8	0/5	0/23	0/4	1.000	
Dry fruit	0/2	0/2	-	-	-	-	
Fruit Candy	0/4	0/1	-	-	0/3	1.000	
Fruit Jam	0/4	0/2	-	-	0/2	1.000	
Kidney Bean	0/7	0/5	0/2	-	-	1.000	
Lentil	4/17	2/6	2/11	-	-	0.495	
Mixed Cereal	1/25	0/2	0/2	0/10	1/11	0.736	
Mixed Seeds	0/12	0/8	0/1	0/1	0/2	1.000	
Oats	4/5	4/5	-	-	-	-	
Others	1/14	0/4	1/3	-	0/7	0.160	
Peanuts	1/4	1/4	-	-	-	-	
Quinoa	0/10	0/5	0/1	0/1	0/3	1.000	
Rice	1/24	1/6	0/2	0/9	0/7	0.392	
**Total**	**18/200**	**12/71**	**4/36**	**0/45**	**2/48**	**0.010**	**1a vs. 2a *p* = 0.012**

* Kruskal-Wallis *p* < 0.001; Multiple comparisons: *p* < 0.001: mixed seeds vs. oats, quinoa vs. oats, chocolate vs. oats, corn vs. oats; *p* = 0.001: chickpeas vs. oats; *p* = 0.002: corn vs. buckwheat.

**Table 3:** Proportion of items containing >20 ppm of gluten by food category (contaminated/tested products).

Food Category	Overall *	Group 1		Group 2		*p*	*p*
		Naturally Gluten Free Products		Products Labeled as “Gluten Free”			
		Group 1a	Group 1b	Group 2a	Group 2b	Kruskal-Wallis	Multiple Comparisons
Breakfast	0/11	0/4	0/1	0/4	0/2	1.000	
Lunch/dinner	15/88	10/45	4/25	0/12	1/6	0.348	
Snacks	2/95	2/22	0/10	0/28	0/35	0.082	
Bread	0/3	-	-	0/1	0/2	1.000	
Pizza	1/3	-	-	-	1/3	-	
Total	18/200	12/71	4/36	0/45	2/48	0.010	1a vs. 2a *p* = 0.012

* Kruskal-Wallis *p* = 0.004; Multiple comparisons: Lunch/dinner versus snacks: *p* = 0.006.
